# Induction of artificial cancer stem cells from tongue cancer cells by defined reprogramming factors

**DOI:** 10.1186/s12885-016-2416-9

**Published:** 2016-07-27

**Authors:** Koji Harada, Tarannum Ferdous, Dan Cui, Yasuhiro Kuramitsu, Takuya Matsumoto, Eiji Ikeda, Hideyuki Okano, Yoshiya Ueyama

**Affiliations:** 1 Department of Oral and Maxillofacial Surgery, Yamaguchi University Graduate School of Medicine, 1-1-1, Minamikogushi, Ube, 755-8505 Japan; 2 Department of Pathology, Yamaguchi University Graduate School of Medicine, 1-1-1, Minamikogushi, Ube, 755-8505 Japan; 3 Department of Biochemistry and Functional Proteomics, Yamaguchi University Graduate School of Medicine, 1-1-1, Minamikogushi, Ube, 755-8505 Japan; 4 Department of Physiology, Keio University, School of Medicine, 35 Shinanomachi, Shinjuku-ku, Tokyo, 160-8582 Japan

**Keywords:** Artificial CSC, Reprogramming factor, Episomal vector, Tongue cancer

## Abstract

**Background:**

The cancer stem cells (CSCs), a small subpopulation of cells in tumor are responsible for the tumor initiation, growth, recurrence and metastasis of cancer, as well as resistance of cancers to drugs or radiotherapy. CSCs are an important target for the development of novel strategies in cancer treatment. However, CSCs-targeted new anti-cancer drug discovery is currently hindered by the lack of easy and reliable methods for isolating, collecting and maintaining sufficient number of CSCs. Here, we examined whether introduction of defined reprogramming factors (*Oct4, shp53, Sox2, Klf4, l-Myc* and *Lin28*) into HSC2 tongue cancer cells could transform the HSC2 into HSC2 with CSCs properties.

**Methods:**

We introduced the defined reprogramming factors into HSC2 tongue cancer cells via episomal vectors by electroporation method to generate transfectant cells. We investigated the malignant properties of the transfectant cells by cell proliferation assay, migration assay, wound healing assay, sphere formation assay, chemosensitivity and radiosensitivity assay in vitro; and also examined the tumorigenic potential of the transfectants in vivo.

**Results:**

The transfectant cells (HSC2/hOCT3/4-shp53-F, HSC2/hSK, HSC2/hUL, HSC2/hOCT3/4-shp53-F + hSK, HSC2/hOCT3/4-shp53-F + hUL, HSC2/hSK + hUL, HSC2/hOCT3/4-shp53-F + hSK + hUL) displayed a malignant phenotype in culture and form tumors on the back of nude mice more efficiently than parental HSC2 and control HSC2/EGFP transfectant cells. They exhibited increased resistance to chemotherapeutic agents; 5-fluorouracil, cisplatin, docetaxel, trifluorothymidine, zoledronic acid, cetuximab, bortezomib and radiation when compared with HSC2 and HSC2/EGFP. Among all the transfected cells, HSC2/hOCT3/4-shp53-F + hSK + hUL cell containing all of the reprogramming factors showed the most aggressive and malignant properties and presented the highest number of spheres in the culture medium containing human recombinant fibroblast Growth Factor-2 (FGF-2) and epidermal Growth Factor (EGF).

**Conclusion:**

These findings suggest that artificial cancer stem cells obtained by the induction of cellular reprogramming may be useful for investigating the acquisition of potential malignancy as well as screening the CSCs-targeting drugs.

## Background

Head and neck squamous cell carcinoma (HNSCC), including oral squamous cell carcinoma (OSCC) is a major health problem throughout the world. Despite advances in treatment of HNSCC, mortality from this disease remains high because of local and regional recurrences of tumors, acquired resistance of patients to chemotherapy or radiotherapy and high rate of metastases at the advanced stage of the disease [1]. Therefore, understanding the initial steps of the tumorigenic processes, as well as tumor progression and metastasis is required for the improvement of prognosis of OSCC and survival of the patients [2].

There are accumulating evidences that cancer cells are functionally heterogeneous and they go through proliferation, differentiation and maturation to a certain extent [3]. A solid tumor contains a distinct subpopulation of cells with stem cell properties (CSCs) that play important roles in cancer initiation, progression, recurrence and metastasis [4–7]. These CSCs are capable of long-term self-renewal and can generate phenotypically diverse tumor cell populations. In addition, they show resistance to radiation and chemotherapeutic agents [8–10]. Therefore, the development of new strategies for CSCs-targeted therapy has attracted attention in these years. Identification and characterization of CSC populations are important for the development of novel strategies for cancer treatment. Separation or generation of a sufficient number of CSCs from tumor tissues and amplification of the CSCs while stably maintaining them in an undifferentiated state in vitro is a pre-requisite to ensure a large-scale drug screening process [11]. CSCs could be identified and isolated through functional assays such as detection of the expression of the cell surface markers specific for CSCs or the sphere body formation method [12–14]. Until now, a number of CSC markers are identified in HNSCC, eg - CD44, CD44v4, CD44v6, ALDH1, CD166, and CD133 [15–19]. There are several other CSC markers reported in different cancer types, eg- CD34, CD133, CD90, CD13, EpCAM, ABCG, ALDH1 etc. [8]. Moreover, Side Population (SP) cells, which are a subpopulation of tumor cells with increased efflux capacity are also considered as a source of CSCs specially where CSC molecular markers are unknown [20, 21]. However, these methods that are dependent upon the isolation of CSCs from tumor tissues by flow cytometry or generation of spheres bodies have some real limitations [12–14]. Therefore, the isolation of sufficient number CSCs from tumor tissues is still difficult. Until now, an easy, cost-saving and optimal cell culture method for successfully amplifying and maintaining pure CSC populations is also unknown. Thus, screening of CSCs-targeting drugs in vitro and in animal models remain in a difficult situation. The development of an easy and efficient method to manufacture artificial CSCs is crucial to overcome these research obstacles.

It is believed that CSCs undergo differentiation into non-CSCs (cancer progeny cells), and that CSCs reach to the top of hierarchical system composed of cancer cells [22–25]. If non-CSCs can be dedifferentiated, CSCs may be generated artificially. Like normal pluripotent stem cells, CSCs are long-lived, and display quiescent potentials in a dormant state, and are responsible for angiogenic induction, apoptotic resistance and differentiation. CSC cells express stem cell marker genes, including *Oct4, Sox2, Nanog, c-kit, ABCG2*, and *ALDH* [3, 26–28]. Among these genes, *Oct4* is the key transcription factor that maintains the pluripotency and self-renewal in undifferentiated embryonic stem cells [27]. Additionally, the essential role of *c-Myc* and *Klf4* in the regulation and maintenance of the stem cell-like features of tumor CSCs was reported by Wang et al. and Yu et al. respectively [29, 30].

The well-known work of generation of induced pluripotent stem cells (iPSCs) by Takahashi and Yamanaka showed that adult somatic cells can be reprogrammed to become pluripotent by the introduction of the pluripotent stem cell genes *Oct4*, *Sox2*, *Klf4* and *c-Myc* [31, 32]. Additionally, Okita et al. mentioned the importance *Lin28* and *l-myc* for the generation of human iPSCs from blood cells [33, 34]. The iPSCs development process shares many features with cancer development. Such similarities indicate that iPSCs reprogramming processes and carcinogenesis might be promoted by overlapping mechanisms; during which, somatic differentiated cell undergoes transcriptional changes and acquires self-renewal and unlimited proliferation capabilities [35–37]. Ohnishi et al. showed that, somatic cells that deviated successful reprogramming failed to develop iPSCs, but behaved similarly to cancer cells and developed Wilms tumor, a childhood blastoma in the kidney [38]. Thus, the same reprogramming factors that generate iPSCs could be also involved in carcinogenic transformation of normal somatic cells. Additionally, in neurosphere culture conditions, introduction of *Oct4, Sox2, c-Myc* and *Klf4* directly induced neural stem cells (NSCs) properties in somatic cells such as skin fibroblasts, which suggests that these reprogramming factors might possess the ability to induce stemness in somatic cells [39–42].

In this study, we followed the iPSCs-generation protocol obtained from the Center for iPS cell research and application (CiRA) website to reprogram HSC2 tongue cancer cells into CSCs [43]. We introduced *l-Myc* instead of *c-Myc* and two other factors (*shp53* and *Lin28*) along with *Oct4, Sox2* and *Klf4* into HSC2 cells via episomal vector; instead of using only *Oct4, Sox2, Klf4* and *c-Myc* with retroviral vectors as initially described by Takahashi and Yamanaka [31–33, 43]. The resultant cells possess the hallmarks of CSCs and could efficiently generate tumors in a nude mouse model. These results suggest that introduction of defined reprogramming factors can possibly dedifferentiate oral cancer cells into CSCs and can provide a potentially valuable system for the study of CSCs.

## Methods

### Cell culture

HSC2 cells were purchased from Cell Bank, RIKEN BioResource Center (Ibaraki, Japan). Cells were cultured in a 1:1 mixture of Dulbecco’s modified Eagle’s medium (D-MEM)/Ham’s F-12 (Wako Pure Chemical Industries, Ltd. Osaka, Japan) supplemented with 10 % fetal bovine serum (FBS) (Thermo Fisher scientific Inc., Waltham, MA, USA), 100 μg/ml streptomycin, 100 units/ml penicillin (Thermo Fisher scientific) in a humidified atmosphere containing 5 % CO_2_ at 37 °C. The electroporated cells, ie - HSC2/EGFP, HSC2/hOCT3/4-shp53-F, HSC2/hSK, HSC2/hUL, HSC2/hOCT3/4-shp53-F + hSK, HSC2/hOCT3/4-shp53-F + hUL, HSC2/hSK + hUL, HSC2/hOCT3/4-shp53-F + hSK + hUL were cultured in the same culture medium without any selection agents.

### Cell reprogramming and transfection

Episomal vectors (pCXLE-hOCT3/4-shp53-F, pCXLE-hSK, pCXLE-hUL and pCXLE-EGFP) were obtained from Addgene (Cambridge, MA, USA) and introduced into HSC2 cells in various combinations. An expression plasmid mixture containing one or more of these episomal vectors (1 μg of each vector) were electroporated into 6 × 10^5^ HSC2 cells with Neon Transfection System (Thermo Fisher scientific) using a 100 μl kit according to the manufacturer’s instructions (conditions for electroporation: pulse voltage: 1550 or 1650 V, pulse width: 10 ms, pulse number: 3). In the same way, we inserted pCXLE-EGFP only into HSC2 cells to obtain HSC2/EGFP as a control. The list of expression plasmid mixtures used in the experiments and the resultant cells is shown in Table 1.

**Table 1:** Summary of plasmid mixtures for electroporation

Mix-ture	Plasmid name	Amount (μg)	Genes	Resultant cell
1	pCXLE-EGFP	1	*Egfp*	HSC2/EGFP
2	pCXLE-hOCT3/4-shp53-F	1	*Oct4*, *shp53, Egfp*	HSC2/hOCT3/4-shp53-F
	pCXLE-EGFP	1		
3	pCXLE-hSK	1	*Sox2*, *Klf4*, *Egfp*	HSC2/hSK
	pCXLE-EGFP	1		
4	pCXLE-hUL	1	*l-Myc*, *Lin28*, *Egfp*	HSC2/hUL
	pCXLE-EGFP	1		
5	pCXLE-hOCT3/4-shp53-F	1	*Oct4*, *shp53,Sox2*, *Klf4*, *Egfp*	HSC2/hOCT3/4-shp53-F + hSK
	pCXLE-hSK	1		
	pCXLE-EGFP	1		
6	pCXLE-hOCT3/4-shp53-F	1	*Oct4, shp53, l-Myc*, *Lin28*, *Egfp*	HSC2/hOCT3/4-shp53-F + hUL
	pCXLE-hUL	1		
	pCXLE-EGFP	1		
7	pCXLE-hSK	1	*Sox2*, *Klf4, l-Myc*, *Lin28*, *Egfp*	HSC2/hSK + hUL
	pCXLE-hUL	1		
	pCXLE-EGFP	1		
8	pCXLE-hOCT3/4-shp53-F	1	*Oct4*, *shp53, Sox2*, *Klf4, l-Myc*, *Lin28*, *Egfp*	HSC2/hOCT3/4-shp53-F + hSK + hUL
	pCXLE-hSK	1		
	pCXLE-hUL	1		
	pCXLE-EGFP	1		

### Cell proliferation assay

HSC2 or each transfectant (5 × 10^3^ cells per well) were seeded on 96-well plates (Becton Dickinson Labware, Franklin lakes, NJ, USA) in D-MEM/Ham’s F-12 medium supplemented with 10 % FBS and 1 % penicillin/streptomycin. After 48 h or 72 h, 3-(4, 5-dimethylthiazol- 2-yl)-2, 5- diphenyltetrazolium bromide (MTT; Sigma-Aldrich, St. Louis, MO, USA) was added to each well (25 μl/well) and incubated for 4 h. Then dimethyl sulfoxide (100 μl/well) was added to each well and a spectrophotometer (BioRad Laboratories, Hercules, CA, USA) was used to measure the absorbance at 490 nm (Optical Density 490 or OD490). All assays were run in triplicate.

### Cell migration assay

Cell migration assay was performed using a Boyden chamber according to the manufacturer’s instructions, (Neuro Probe, Gaithersburg, MD, USA). 5 × 10^3^ cells in 50 μl D-MEM/Ham’s F-12 medium without FBS were seeded on a gelatin coated polycarbonate membrane. In the lower chamber, 25 μl D-MEM/Ham’s F-12 with 10 % FBS was added as chemoattractant. After the cells were incubated for 24 h at 37 °C in a 5 % CO_2_ atmosphere, the polycarbonate membrane was washed with PBS, and cells on the top surface of the polycarbonate membrane were removed with a cotton swab. Cells adhering to the lower surface were fixed with methanol, stained with Hematoxylin solution and counted under a microscope in five predetermined fields (200×). All assays were independently repeated at least three times.

### Wound healing assay

Cells (15 × 10^3^ cells per well) were seeded into 24-well plate (Becton Dickinson Labware) and were cultured in D-MEM/Ham’s F-12 with 10 % FBS and 1 % penicillin/streptomycin until a monolayer of cells were formed. A 200 μl pipette tip was used to gently wound cell layer through the central axis of the plate. The migration of cells into the wounded area was observed at 24 h by a microscope (BX-51-33-FLD2, OLYMPUS, PA, USA). The cell wound closure rate was calculated using the following equation: Wound closure = [1 − (wound area at Tt/wound area at T0) × 100, where Tt is the time passed since wounding (24 h) and T0 is the time of initial wounding. The experiments were performed in triplicate.

### Tumor sphere formation assay

Cells were placed at a density of 1000 cells/well in sphere-culture medium consisting of serum-free DMEM/F-12 (Ham) 1:1 (Prototype) medium (Thermo Fisher scientific), N2 supplement (Thermo Fisher scientific), 10 ng/ml FGF-2 (Thermo Fisher scientific), and 10 ng/ml EGF (Thermo Fisher scientific) in a ultra-low attachment 96-well plate (Corning, New York, NY, USA) to generate primary sphere bodies. Every four days, 50 μL of the fresh growth medium was added. The numbers of spheres larger than 20 μm in diameter were counted after two weeks of culture.

### Western blotting

Whole cell lysates were prepared using Radioimmunoprecipitation assay (RIPA) buffer (Thermo Fisher scientific) and were subjected to electrophoresis on 10 % SDS-polyacrylamide gels (Thermo Fisher scientific), and then transferred to a PVDF membrane (Thermo Fisher scientific). After blocking, the membranes were incubated with the anti-HCAM (CD44) mouse monoclonal antibody (Santa Cruz Biotechnology, Inc., Santa Cruz, CA, USA) and anti-CD13 rabbit monoclonal antibody (Epitomics Inc., Burlingame, CA, USA) followed by Novex® alkaline-phosphatase conjugated (goat) anti-rabbit or (goat) anti-mouse immunoglobulin G (IgG) secondary antibody (Thermo Fisher scientific). The antibodies were detected using a chromogenic immunodetection system, WesternBreeze (Thermo Fisher scientific) according to the manufacturer's instructions. Also, anti- α- tubulin monoclonal antibody (Santa Cruz Biotech.) was used for normalization of Western blot analysis.

### Chemosensitivity and radiosensitivity assessment

Cells (5 × 10^3^ cells per well) were seeded on 96-well plates (Becton Dickinson Labware) in D-MEM/Ham’s F-12 with 10 % FBS and 1 % penicillin/streptomycin. Twenty four hours later, cells were either remained untreated or were treated with any one of the following drugs: 2 μg/ml 5-fluorouracil (5-FU), 1 μg/ml cisplatin (CDDP), 100 pg/ml docetaxel (DOC), 50 μg/ml trifluorothymidine (TFT), 1 μg/ml cetuximab, 5 ng/ml bortezomib or 10 μg/ml zoledronic acid. Cells were also exposed to 15 Gy radiation in an X-ray irradiator (MBR-1505R2, 150 kV, 5 mA, filter: 1.0 mm aluminum, Hitachi Medico, Tokyo, Japan). After 48 h, 25 μl MTT was added to each well. After 4 h, dimethyl sulfoxide (100 μl/well) was added and the absorbance was measured with a spectrophotometer (BioRad Laboratories) at 490 nm. All assays were run in triplicate.

### In vivo tumor formation assay

Cells (1 × 10^4^ ~ 1 × 10^6^) were washed twice with antibiotic-free and serum-free D-MEM/Ham’s F-12 medium and finally re-suspended in 0.1 ml of saline. The cell suspension was injected subcutaneously into 5-week-old BALB/c nude mice (CLEA, Tokyo Japan). Tumor size was monitored and measured weekly for 4 weeks. The estimated tumor volume was calculated as 0.5 × length × width^2^. All mice were sacrificed at the end of 4 weeks/28 days. The tumors were dissected out, fixed in neutral-buffered formalin, embedded in paraffin and stained with hematoxylin and eosin. The mice were housed in a pathogen-free environment under a 12 h light/dark cycle, and provided with sterile water and food ad libitum. All studies and experiments conformed to the Guidelines for Animal Experimentation of Yamaguchi University (Ube, Japan).

## Results

### Establishment of transfectants by defined reprogramming factors

**Fig. 1:**
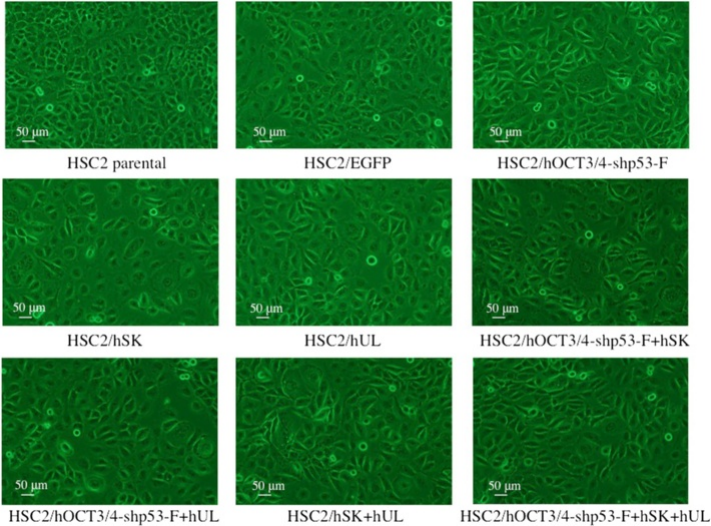
Cell morphology. All transfectants are morphologically distinct from their parental cell line (HSC2) and from each other. All transfectants have shown the loss of cell-cell adhesion (except HSC2/EGFP) and showed spindle-shaped morphology

We successfully introduced the reprogramming factors (*Oct4, shp53, Sox2, Klf4, l-Myc* and *Lin28*) and *Egfp* genes via the plasmid vectors (pCXLE-hOCT3/4-shp53-F, pCXLE-hSK, pCXLE-hUL and pCXLE-EGFP) into HSC2 cells by electroporation in order to obtain HSC2/EGFP, HSC2/hOCT3/4-shp53-F, HSC2/hSK, HSC2/hUL, HSC2/hOCT3/4-shp53-F + hSK, HSC2/hOCT3/4-shp53-F + hUL, HSC2/hSK + hUL and HSC2/hOCT3/4-shp53-F + hSK + hUL cells. Fluorescence microscopic observation of EGFP expression in transfectant cells showed the vector transplantation efficiency was about 50 % when the pulse voltage of the electroporator was 1650 V, and that about 30 % at 1550 V (data not shown). Therefore, the optimum condition for electroporation was set as; pulse voltage: 1650 V, pulse width: 10 ms, pulse number: 3. The transfectants were cultured in D-MEM/Ham’s F-12 medium supplemented with 10 % FBS, 1 % penicillin/streptomycin. In this study, we did not use any selection methods to identify stable transfectants. The transfected cells were bigger and spindle-shaped compared to HSC2 parental cells which had cobblestone morphology (Fig. 1) Moreover, each transfectant cells showed slightly different morphology than the other (Fig. 1).

### Cell proliferation ability

**Fig. 2:**
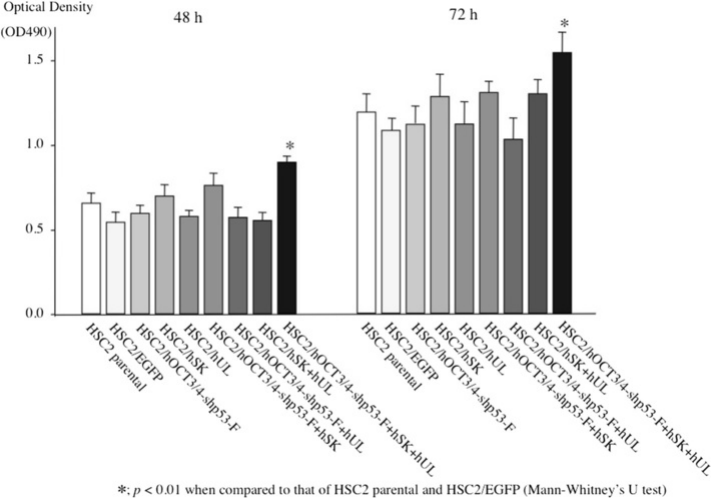
Cell proliferation assay. Cells (5 × 10^3^ cells per well) were seeded on 96-well plates, and cultured for 48 and 72 h. Cell growth was evaluated by MTT assay (OD490; absorbance at 490 nm). The growth rate of HSC2/hOCT3/4-shp53-F + hSK + hUL was significantly higher than that of HSC2 and HSC2/EGFP at 48 and 72 h after seeding. Error bars represent the standard deviation of the mean of three independent experiments

MTT assay was used to measure the growth rate of the transfectants. The HSC2/hOCT3/4-shp53-F + hSK + hUL had higher proliferative ability than that of parental cell (HSC2) and HSC2/EGFP. At 48 h and 72 h of culture, the growth rate of HSC2/hOCT3/4-shp53-F + hSK + hUL was significantly higher than that of HSC2 and HSC2/EGFP, while the growth rate among parental HSC2 cell, HSC2/EGFP and the other transfectants was not significantly different (Fig. 2).

### Migration ability

**Fig. 3:**
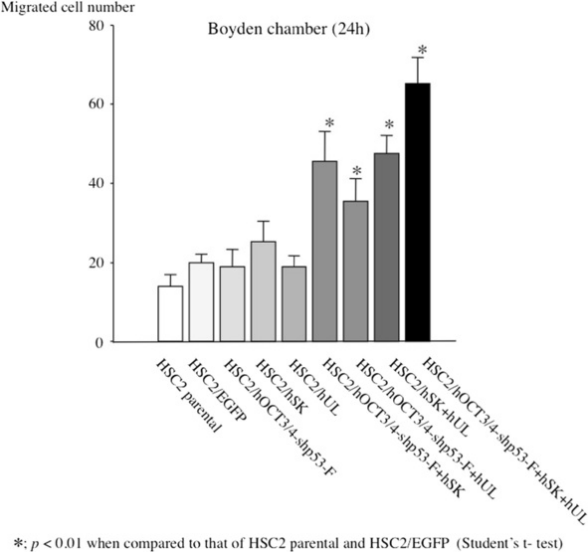
Migration assay. To evaluate the migration activity for the transfectants, migration assay was performed with Boyden chamber. The HSC2/hOCT3/4-shp53-F + hSK + hUL showed highest migration ability. Error bars represent the standard deviation of the mean of three independent experiments

CSCs are endowed with high migratory ability. Therefore, we measured the migration activity of the transfectants using migration assay with Boyden chamber. The HSC2/hOCT3/4-shp53-F + hSK, HSC2/hOCT3/4-shp53-F + hUL, HSC2/hSK + hUL and HSC2/hOCT3/4-shp53-F + hSK + hUL had significantly higher migration ability than that of parental cell (HSC2) and HSC2/EGFP. Especially, the HSC2/hOCT3/4-shp53-F + hSK + hUL showed highest migration ability (Fig. 3).

### Wound healing ability

**Fig. 4:**
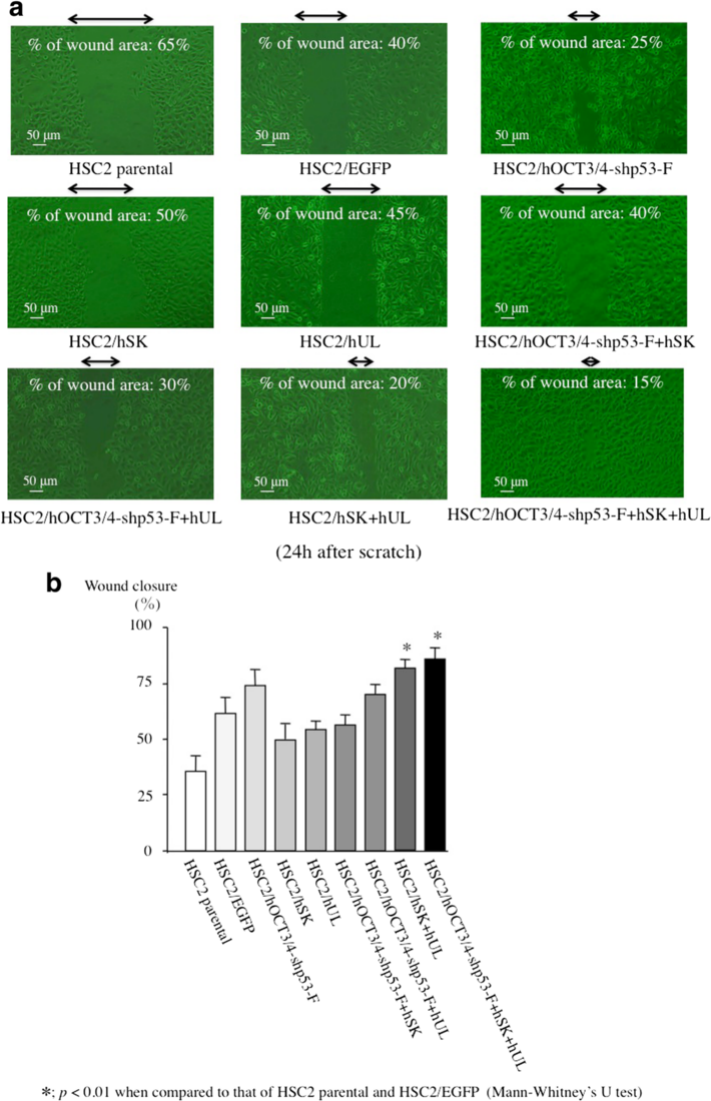
Wound healing assay. The wound healing ability for the transfectants was measured by wound healing assay. A. The HSC2/hOCT3/4-shp53-F + hSK + hUL had higher wound healing ability than that of HSC2 and HSC2/EGFP. Arrows show the width of uncovered scratch mark. The wound area was calculated according to the following formula: (wound area at Tt/wound area at T0) × 100, where Tt is the time passed since wounding and T0 is the time of initial wounding. B. The wound healing capacity for each cell type was calculated using the following equation: Wound closure = [1‑(wound area at Tt/wound area at T0) × 100. Error bars represent the standard deviation of the mean of three independent experiments

Wound healing assay was performed to examine the migration capability of the transfectants. HSC2/hOCT3/4-shp53-F + hSK + hUL cell showed highest would healing capacity (wound closure: 85 %) compared to HSC2/EGFP (60 %), all the other transfectants (50–80 %) and the parental HSC2 cell (35 %). Interestingly, all the transfectant cells showed higher wound healing ability than that of parental HSC2 (Fig. 4a, b). HSC2/hUL + hSK cells showed second highest migration and wound healing capacity (80 %) which suggests that *Sox2, Klf4, l-Myc, Lin28* factors are essential to increase the migration capacity of the transfectants.

### Sphere formation

**Fig. 5:**
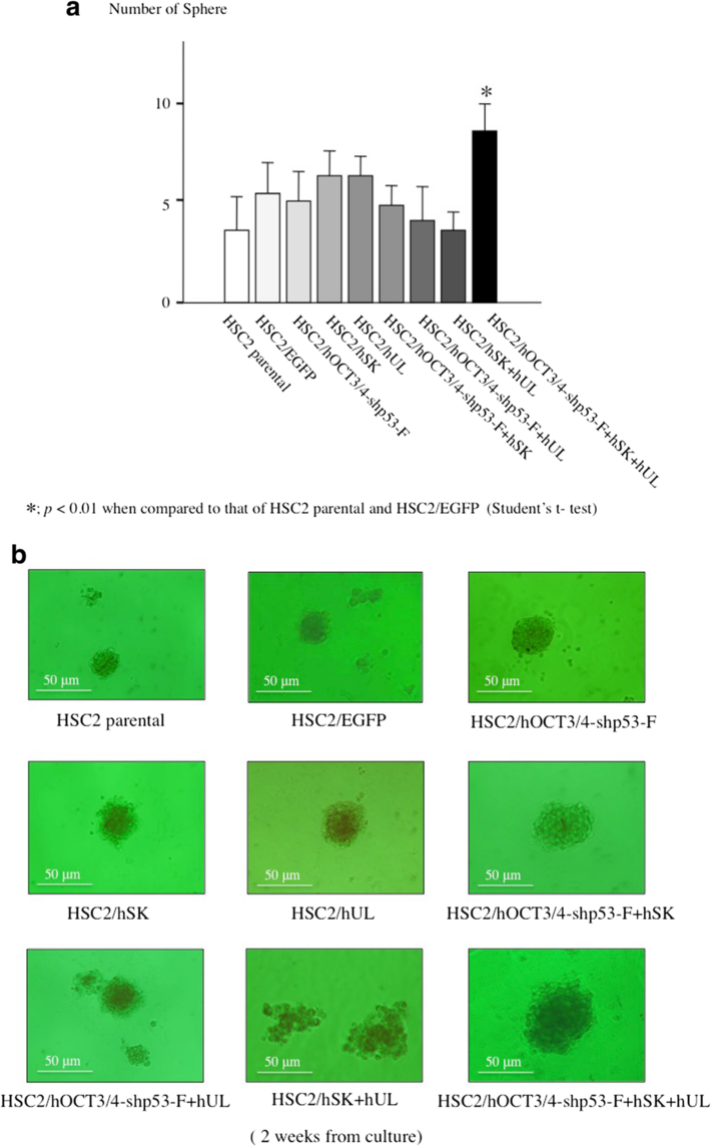
Sphere formation assay. The transfectants and HSC2 cells were cultured in a sphere-culture medium. **a** The number of spheres was significantly higher in the HSC2/hOCT3/4-shp53-F + hSK + hUL than HSC2 and HSC2/EGFP. Error bars represent the standard deviation of the mean of three independent experiments. **b** Representative images of sphere (s) generated from each cell types (Bar  =  50 μm). The HSC2/hOCT3/4-shp53-F + hSK + hUL could also form bigger spheres

The transfectants, HSC2/EGFP and HSC2 cells were cultured in tumor sphere-culture medium. All cells were able to grow and form primary spheres within 2 weeks of culture; however, the HSC2/hOCT3/4-shp53-F + hSK + hUL cells could form spheres burly and numerously. Briefly, the number of spheres was significantly higher in the HSC2/hOCT3/4-shp53-F + hSK + hUL cell than HSC2 and HSC2/EGFP cell (Fig. 5a). HSC2/hOCT3/4-shp53-F + hSK + hUL cells also developed comparatively bigger spheres than other cell types (Fig. 5b).

### Expression of CSC markers

**Fig. 6:**
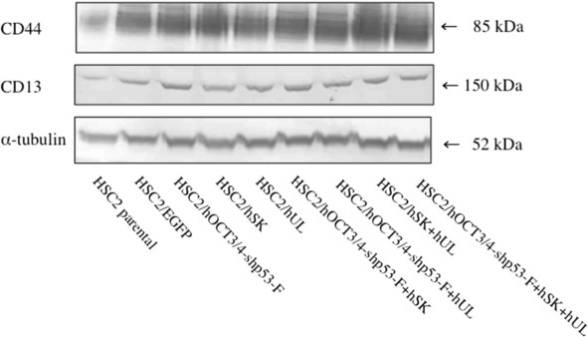
Expression of CSC markers. Western blotting was performed to investigate protein levels of CSC markers (CD44 and CD13). CD44 expression was significantly higher in HSC2/hOCT3/4-shp53-F + hSK + hUL than other transfectants and HSC2. CD13 expression was higher in all other transfectant cells than HSC2/EGFP and parental HSC2. α-tubulin was used as an internal control

The expression pattern of CSC markers (CD44 and CD13) in the transfectants and HSC2 cells was analyzed by Western blotting. The protein expression of CD44 was significantly higher in HSC2/hOCT3/4-shp53-F + hSK + hUL than in HSC2/EGFP and HSC2, whereas CD13 expression was higher in all transfectant cells compared to HSC2 parental cell (Fig. 6).

### Chemosensitivity and radiosensitivity (in vitro)

**Fig. 7:**
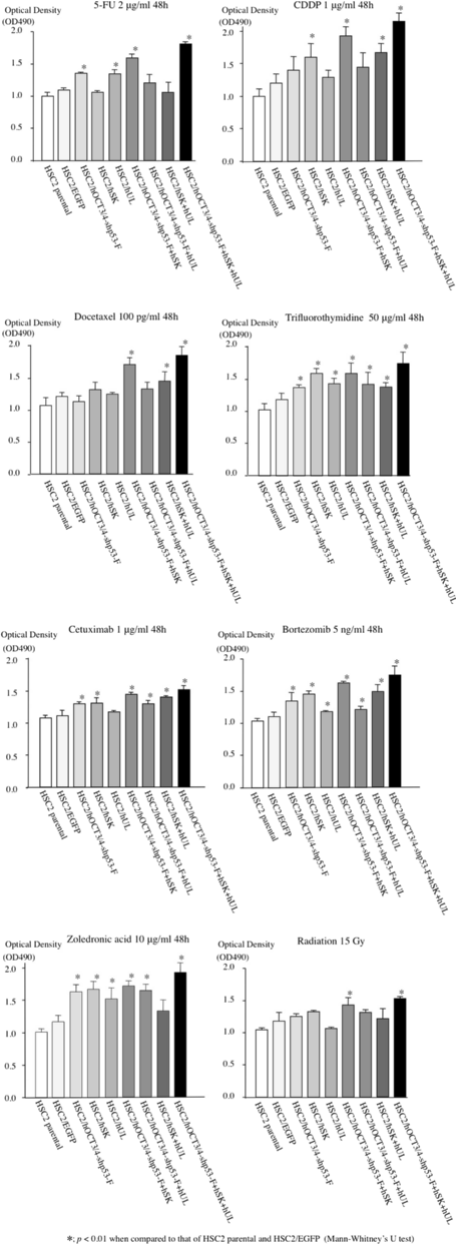
Chemosensitivity and radiosensitivity assay. To evaluate the resistance of the transfectants to various chemotherapeutic drugs, molecular-targeted agents, and radiation, MTT assay was performed (OD490; absorbance at 490 nm). The HSC2/hOCT3/4-shp53-F + hSK + hUL was significantly more resistant to 5-FU, CDDP, DOC, TFT, zoledronic acid, cetuximab, bortezomib and X-ray radiation than HSC2 and HSC2/EGFP. Error bars represent the standard deviation of the mean of three independent experiments

CSCs show resistance to anticancer drugs and radiation. Therefore, we evaluated the resistance ability of the transfectants to several chemotherapeutic drugs and radiation. The HSC2/hOCT3/4-shp53-F + hSK + hUL cell showed increased resistance to 5-FU, CDDP, DOC, TFT, zoledronic acid, cetuximab, bortezomib and X-ray radiation than other transfectants and HSC2 cells (Fig. 7).

### Tumorigenesis (in vivo)

**Fig. 8:**
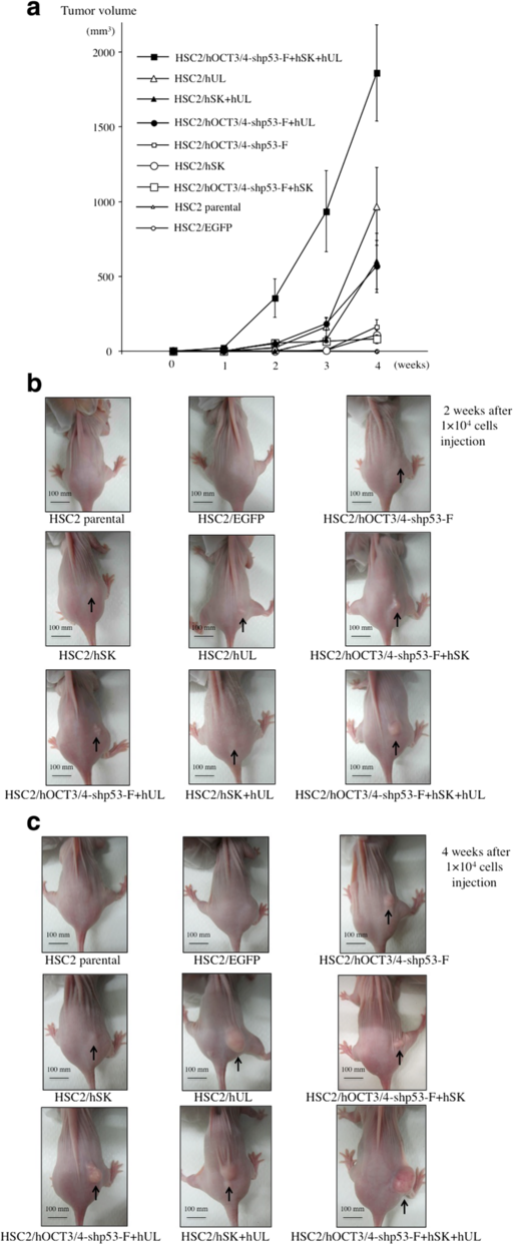
In vivo tumor formation assay. Each transfectant, HSC2 or HSC2/EGFP (1 × 10^4^) were inoculated into BALB/c nude mice subcutaneously and tumor volume was measured once a week for 4 weeks. **a** Change of tumor volume. Error bars represent the standard error of the mean from three mice results (n = 3). **b** Nude mice tumor at 2 weeks after 1 × 10^4^ cells injection. **c** Nude mice tumor at 4 weeks after 1 × 10^4^ cells injection (Bar  =  10 mm). Arrows show the tumor mass

**Fig. 9:**
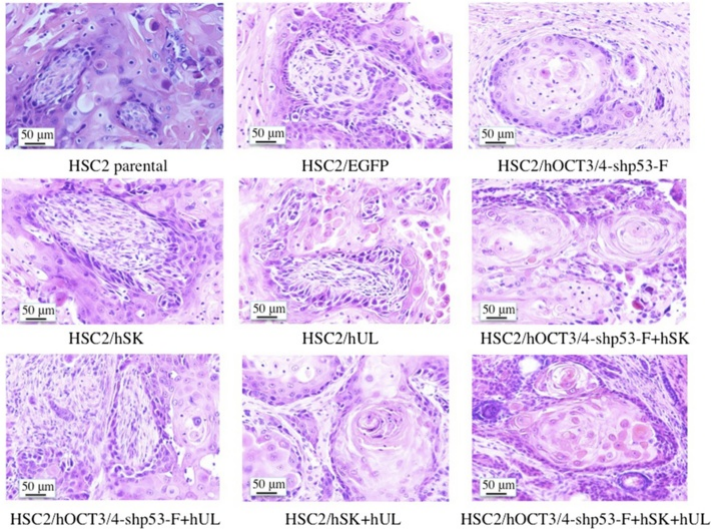
Histological analysis of nude mice tumors. HSC2 or HSC2/EGFP (5 × 10^6^) were inoculated into BALB/c nude mice subcutaneously to make each tumor mass. Each tumor was dissected out, fixed in neutral-buffered formalin, embedded in paraffin and stained with hematoxylin and eosin. Histological analysis of these tumors showed squamous cell carcinoma tissues including from highly to poorly differentiated areas, but teratomas were not observed (Bar  =  50 μm)

The tumorigenic capacity of the transfectants was assessed using a mouse model. We injected each transfectant, HSC2 or HSC2/EGFP subcutaneously into BALB/c nude mice and monitored them for 4 weeks. While 1 × 10^4^ cells of HSC2/hOCT3/4-shp53-F + hSK + hUL were enough to generate tumor in mice within 2 weeks, the same number of cells did not produce any HSC2 or HSC2/EGFP tumors. The minimum number of cells required for generating HSC2 or HSC2/EGFP tumor in mice was 1 × 10^6^ (data not shown), which was 100-fold higher than the number of HSC2/hOCT3/4-shp53-F + hSK + hUL required for tumor seeding. In addition, HSC2/hOCT3/4-shp53-F + hSK + hUL showed higher tumorigenic potential (Fig. 8a–c). Histological analysis of these tumors showed squamous cell carcinoma tissues including from highly to poorly differentiated areas, but teratomas were not observed (Fig. 9).

## Discussion

In this study, we demonstrated that the introduction of defined reprogramming factors (*Oct4, shp53, Sox2, Klf4, l-Myc* and *Lin28*) could generate cells with CSC-like properties from a tongue cancer cell line, HSC2. These artificial CSCs showed resistance to anticancer drugs, molecular target drugs and radiation. Moreover, these artificial CSCs could be maintained in a cost-effective way. D-MEM medium supplemented with 10 % FBS was sufficient to maintain these cells with CSC properties. These artificial CSCs could be handled easily and also managed to produce spheres in sphere-culture medium. Therefore, we may be able to develop a new strategy for generating and maintaining artificial CSCs.

We used HSC2 cell line in our present study, as it has neither invasive nor metastatic potential and showed low expression of the stem cell markers, ie- CD44 and CD13 (Fig. 6). In this study we also examined the expression of CD44v6, ALDH1, CD133 and podoplanin/D2-40, but their expression patterns in transfectant cells were not different compared to that of HSC2 parental cells (data not shown). After introduction of the reprogramming factors, most of the transfectants showed higher invasion and migration ability than HSC2 or HSC2/EGFP. Interestingly, HSC2/hOCT3/4-shp53-F + hSK + hUL cell containing all the reprogramming factors had acquired highest malignant properties in migration assay (Fig. 3), wound healing assay (Fig. 4) and sphere formation assay (Fig. 5) compared to the other transfectants, while demonstrating high expression of CD44. Moreover, our in vivo study showed that tumors developed from HSC2/hOCT3/4-shp53-F + hSK + hUL cells containing all the reprogramming factors (*Oct3/4*, *shp53, Sox2*, *Klf4*, *l-Myc* or *Lin28*) exhibited higher tumorigenic potential than parental HSC2 cells. Additionally, these tumors were not teratomas which suggest that our transfectant cells are not iPSCs.

CSCs may be derived from tissue stem cell, however, most of CSCs may be generated from non-tumorigenic differentiated epithelial cells by reprogramming. Nishi et al. reported that the introduction of defined reprogramming factors (*Oct4, Sox2, Klf4 and c-Myc*) into MCF-10A nontumorigenic mammary epithelial cells, followed by partial differentiation, transforms the bulk of cells into tumorigenic cells with CSC properties [44]. Miyoshi et al. reported that introduction of defined reprogramming factors (*Oct4*, *Sox2*, *Klf4* and *c-Myc*) into human gastrointestinal cancer cell lines resulted improved sensitivity of the induced cells to chemotherapeutic agents and differentiation-inducing treatment [45]. Moreover, Oshima et al. reported the generation of CSCs with lineage specificity directly from colon cancer cells by introducing same defined factors (except *c-Myc*), not via an induced pluripotent stem cell state [46].

There are many attempts to generate iPSCs from malignant tumor cells by the ectopic expression of reprogramming factors. Carette et al. reported to generate the iPS cells derived from human chronic myeloid leukemia cells by using the reprogramming factors (*Oct4*, *Sox2*, *Klf4* and *c-Myc*) [47]. Similarly, Utikal et al. reported that they attempted to create iPSCs from melanoma by using the reprogramming factors [48]. However, it seems to achieve only limited success. It may be selected whether the ectopic expression of reprogramming factors in malignant tumor cell lines can induce cells with CSC-like properties or iPSCs during the reprogramming step.

CSCs are known to have high proliferation potency in some cancer types (eg - leukemia), whereas other reports describes CSCs as a slowly dividing cell population (eg- melanoma) and it has also been suggested that as CSCs divide slowly, the CSC population is responsible for tumor resistance to treatment [49]. However, our HSC2/hOCT3/4-shp53-F + hSK + hUL cell had higher proliferative activity though it showed resistance to various chemotherapeutic drugs, molecular-targeted agents, and radiation (Figs. 2 and 7). It is the general idea that, higher proliferation potency may lead to higher intake of anticancer agents inside the cancer cell. One proposed model that explains the origin of CSC’s ability to survive conventional chemotherapeutic regimens describes that, only the CSCs overexpressing ATP-binding cassette (ABC) transporters are able to repopulate the tumor after exposure to the chemotherapeutic agents [50]. The multidrug efflux pump ABCG2 has roles in cytotoxic drug efflux and has been described as one of the reason of the “side population” which helps define adult stem cells of tumors [51]. So, we examined the expression of ABCG2 between the transfectants and HSC2/EGFP or HSC2 to investigate the reason why the HSC2/hOCT3/4-shp53-F + hSK + hUL showed resistance to various chemotherapeutic drugs in spite of its higher proliferation potency. However, we could not detect any differences of ABCG2 expression between the transfectants and HSC2/EGFP or HSC2 (data not shown).

The CSC markers differ in different cancer types. Until now, a number of markers have been discovered, ie - CD44, CD44v4, CD44v6, CD34, CD133, CD166, CD90, CD13, EpCAM, ABCG2, ALDH1 etc. [8, a,b,c]. However, in this present study we reported the expression pattern of only a few. Further experimentation is necessary to understand the expression of other CSC markers in HSC2 and in the transfectants. The reprogramming factors (*Oct4*, *Sox2*, *Klf4*, *l-Myc* or *Lin28*) we used in this study might be a few of the key molecules that can trigger the conversion of non-CSCs into CSC in oral tumors. Therefore, it is necessary to identify the other key molecules that are required for the development of CSCs.

## Conclusion

In summary, we here describe the possibility of reprogramming non-CSCs by the introduction of defined reprogramming factors and the consequent generation of artificial CSCs. These findings may provide a valuable model system for the study of CSCs, which might help in the development of new therapeutic strategies targeting CSCs in oral tumors.

## Abbreviations

5-FU, 5-fluorouracil; ABC transporters, ATP-binding cassette transporters; CDDP, cisplatin; CiRA, center for iPS cell research and application; CSC, cancer stem cell; D-MEM, Dulbecco’s modified Eagle’s medium; DOC, docetaxel; EGF, epidermal growth factor; FBS, fetal bovine serum; FGF-2, fibroblast growth factor-2; HNSCC, head and neck squamous cell carcinoma; IgG, immunoglobulin G; iPSC, induced pluripotent stem cell; MTT, 3-(4, 5-dimethylthiazol- 2-yl)-2, 5- diphenyltetrazolium bromide; NSC, neural stem cell; OD: optical density; OSCC, oral squamous cell carcinoma; RIPA, radioimmunoprecipitation assay; TFT, trifluorothymidine
